# Adherence to Prolactin Monitoring Guidelines in Children and Adolescents Prescribed Risperidone: A Clinical Audit From a Tertiary Mental Health Service in the United Arab Emirates

**DOI:** 10.1192/bjo.2026.11691

**Published:** 2026-06-30

**Authors:** Salma Al Alazeezi, Zahra Ahmed, Duha Al Ali, Syed Fahad Javaid

**Affiliations:** 1 Behavioural Sciences Institute, Al Ain, UAE; 2 Department of Psychiatry, College of Medicine and Health Sciences, United Arab Emirates University, Al Ain, UAE

## Abstract

**Aims:**

Risperidone is prescribed to children and adolescents for psychotic conditions when non-drug interventions are insufficient. It carries a significant risk of hyperprolactinaemia, which may adversely affect growth, pubertal development, and bone health. This audit assessed adherence to local hospital guidelines on prolactin monitoring for child and adolescent patients on risperidone, which align with the National Institute for Health and Clinical Excellence (NICE) Guideline CG155, at the Behavioural Sciences Institute (BSI) in Al Ain, United Arab Emirates (UAE).

**Methods:**

A retrospective clinical audit was conducted at the BSI, Al Ain Hospital. Electronic medical records were reviewed for patients aged 18 years or younger who were initiated on risperidone from January 2023 onwards and maintained on treatment for at leastsix months. Data were collected between April and July 2025. Patients who discontinued risperidone within six months or had incomplete follow-up were excluded. Audit standards were based on local hospital protocols aligned with national guidelines, focusing on documentation of prolactin measurements at baseline, 12 weeks, and six months after treatment initiation. Data were analysed descriptively and reported as frequencies and percentages.

**Results:**

Of 52 cases reviewed, 49 met the inclusion criteria. The sample comprised 42 males (86%), with ages ranging from 3 to 17 years (mean 9.6 years). The majority of patients (96%) had diagnoses of psychosis associated with neurodevelopmental conditions, including attention-deficit hyperactivity disorder, autism spectrum disorder, or intellectual disability. Baseline prolactin monitoring was performed more frequently than follow-up monitoring. Prolactin measurement at 12 weeks was documented in 12% of patients, with reasons recorded for non-monitoring in 8% of cases. Monitoring at the six-month interval was more commonly completed, although a substantial proportion of patients did not undergo recommended testing at this time point.

**Conclusion:**

This audit identified partial adherence to prolactin monitoring guidelines in children and adolescents prescribed risperidone at a tertiary mental health service. While baseline and six-month monitoring were more frequently completed, monitoring at the 12-week interval and documentation of reasons for missed tests were suboptimal. These findings highlight the need for targeted quality-improvement strategies, including electronic reminders, staff education, and improved documentation practices, to ensure safer prescribing and early detection of antipsychotic-induced hyperprolactinaemia in this vulnerable population.

No financial sponsorship has been received for this project.

